# Pyrazolo[3,4-*d*]pyrimidine Tyrosine Kinase Inhibitors Induce Oxidative Stress in Patient-Derived Glioblastoma Cells

**DOI:** 10.3390/brainsci11070884

**Published:** 2021-06-30

**Authors:** Ana Kostić, Sofija Jovanović Stojanov, Ana Podolski-Renić, Marija Nešović, Miodrag Dragoj, Igor Nikolić, Goran Tasić, Silvia Schenone, Milica Pešić, Jelena Dinić

**Affiliations:** 1 Department of Neurobiology, Institute for Biological Research “Siniša Stanković”—National Institute of Republic of Serbia, University of Belgrade, Bulevar Despota Stefana 142, 11060 Belgrade, Serbia; ana.kostic@ibiss.bg.ac.rs (A.K.); sofija.jovanovic@ibiss.bg.ac.rs (S.J.S.); ana.podolski@ibiss.bg.ac.rs (A.P.-R.); marija.stepanovic@ibiss.bg.ac.rs (M.N.); miodrag.dragoj@ibiss.bg.ac.rs (M.D.); camala@ibiss.bg.ac.rs (M.P.); 2 Clinic for Neurosurgery, Clinical Center of Serbia, Pasterova 2, 11000 Belgrade, Serbia; i.m.nikolic@gmail.com (I.N.); dr.gorantasic@gmail.com (G.T.); 3 School of Medicine, University of Belgrade, Doktora Subotića 8, 11000 Belgrade, Serbia; 4 Department of Pharmacy, University of Genova, Viale Benedetto XV 3, 16132 Genova, Italy; schenone@difar.unige.it

**Keywords:** glioblastoma, Src tyrosine kinase inhibitor, oxidative stress, anticancer activity

## Abstract

Background: Glioblastoma (GBM) highly expresses Src tyrosine kinase involved in survival, proliferation, angiogenesis and invasiveness of tumor cells. Src activation also reduces reactive oxygen species (ROS) generation, whereas Src inhibitors are able to increase cellular ROS levels. Methods: Pro-oxidative effects of two pyrazolo[3,4-*d*]pyrimidine derivatives—Src tyrosine kinase inhibitors, Si306 and its prodrug pro-Si306—were investigated in human GBM cells U87 and patient-derived GBM-6. ROS production and changes in mitochondrial membrane potential were assessed by flow cytometry. The expression levels of superoxide dismutase 1 (SOD1) and 2 (SOD2) were studied by Western blot. DNA damage, cell death induction and senescence were also examined in GBM-6 cells. Results: Si306 and pro-Si306 more prominently triggered ROS production and expression of antioxidant enzymes in primary GBM cells. These effects were followed by mitochondrial membrane potential disruption, double-strand DNA breaks and senescence that eventually led to necrosis. Conclusion: Src kinase inhibitors, Si306 and pro-Si306, showed significant pro-oxidative potential in patient-derived GBM cells. This feature contributes to the already demonstrated anti-glioblastoma properties of these compounds in vitro and in vivo and encourages clinical investigations.

## 1. Introduction

Brain tumors represent 90% of all tumors of the central nervous system (CNS) [1]. Glioblastoma (GBM) is the most frequent and aggressive (WHO grade IV) type of malignant CNS tumor and is associated with poor prognosis, with a median patient survival of 12 to 15 months [2,3]. The main characteristics of GBM include high proliferation rate, infiltrating nature and resistance to chemotherapy and radiation. The pathogenesis of GBM is very complex and includes alterations of numerous key cellular pathways that regulate cell proliferation, survival, migration and angiogenesis.

In recent years, targeted therapy with drugs directed against molecules with key roles in proliferation, survival and invasiveness of cancer cells came into focus. Tyrosine kinases (TKs), with their primary role in tumorigenesis, have become potential targets for the therapy of several cancer types [4]. Therefore, small molecules that inhibit the kinase domains of specific TKs have been introduced into the clinical practice of a number of solid tumors [5]. Nevertheless, many tyrosine kinase inhibitors did not display adequate efficacy against GBM tumors thus far due to the aggressive and invasive nature of GBM, development of drug resistance and ineffective drug delivery across the blood–brain barrier [6].

The deregulation of Src family tyrosine kinases (SFKs) activity is responsible for the growth and progression of various cancers [7], while elevated Src activity has been reported in GBM [8,9,10]. The discovery of SFKs importance in the biology of glioblastoma has put focus on dasatinib as a new therapeutic. This potent SFK inhibitor decreases the autophosphorylation of Src kinase and consequently reduces downstream signaling from Src to Akt in GBM cell lines [11], inhibiting their proliferation and invasive potential [12,13]. However, the failure of its application is reflected in unfavorable pharmacokinetic properties, such as short half-life and insufficient delivery to the CNS tumors.

One of the Src tyrosine kinase regulation mechanisms is via reactive oxygen species (ROS) [14]. However, the mechanisms through which a redox-sensitive Src kinase responds to ROS have not been entirely determined [14]. A study reported that Src activation attenuated ROS generation, whereas on the contrary Src inhibitors saracatinib and SU6656 increased cellular ROS levels [15]. Other tyrosine kinase inhibitors such as dasatinib, imatinib and sunitinib have been shown to increase mitochondrial ROS levels and decrease the mitochondrial membrane potential, leading to cell death [16,17].

**Figure 1:**
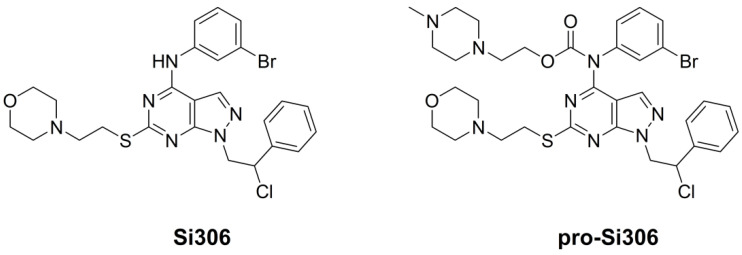
Molecular structure of Si306 and pro-Si306.

Novel TK inhibitors with pyrazolo[3,4-*d*]pyrimidine scaffold are ATP-competitive TK inhibitors with good efficacy regarding the inhibition of SFKs [18,19], and their activity against neuroblastoma and glioblastoma has been demonstrated in vitro and in vivo [20,21,22,23,24,25]. Specifically, a pyrazolo[3,4-*d*]pyrimidine derivative, Si306 (Figure 1), strongly suppressed U87 glioblastoma xenograft growth in nude mice in combination with radiotherapy, and the findings indicated efficient delivery of Si306 to the brain [26]. Although prodrugs often have significantly lower biological activity compared to parent drugs [27], comparable results were obtained with its prodrug, pro-Si306 (Figure 1), which displayed improved pharmacological properties compared to Si306. We demonstrated that pro-Si306 possesses enhanced solubility and efficiency in orthotopic GBM model compared to its parent drug [26].

In addition, our earlier studies showed that Si306 and pro-Si306 were twice as efficient in inhibiting GBM cell growth compared to dasatinib and were equally effective in sensitive and multidrug-resistant GBM cells [20,22,25]. We showed that Si306 and pro-Si306 suppress the activity of P-glycoprotein, a membrane efflux transporter whose overexpression is responsible for glioblastoma resistance to therapy and has an important role in the blood–brain barrier. Si306 and its prodrug also significantly reduced the invasive potential of several primary GBM cultures, as well as GBM xenografts in zebrafish embryo model [25].

Our previous findings encouraged the further investigations of these pyrazolo[3,4-*d*]pyrimidine derivatives as potential candidates for new targeted therapy of glioblastoma. In this study, we aimed to evaluate the pro-oxidative effect of Si306 and pro-Si306 in GBM cells.

## 2. Materials and Methods

### 2.1. Drugs

Derivatives of pyrazolo[3,4-*d*]pyrimidine, Si306 and its prodrug pro-Si306, were obtained as previously described [26]. Dasatinib was purchased from Sigma-Aldrich Chemie GmbH (Taufkirchen, Germany). Si306, pro-Si306 and dasatinib were dissolved in dimethyl sulfoxide (DMSO) and kept at room temperature as 20 mM aliquots. Immediately before treatment, drugs were diluted in sterile phosphate buffered saline (PBS).

### 2.2. Chemicals

Minimum Essential Medium (MEM) was purchased from Capricorn Scientific GmbH (Ebsdorfergrund, Germany), while DMEM/F12 medium, penicillin-streptomycin solution, amphotericin B solution, L-glutamine and trypsin were purchased from Biowest (Nuaillé, France). Fetal bovine serum (FBS), non-essential amino acids, DMSO, Thiazolyl blue tetrazolium bromide (MTT) and JC-1 were obtained from Sigma-Aldrich Chemie GmbH. Dihydroethidium (DHE), dihydrorhodamine 123 (DHR), fluorescein-di-β-D-galacto-pyranoside (FDG) and hoechst 33342 were purchased from Thermo Fisher Scientific (Waltham, MA, USA). Annexin-V-FITC (AV) and propidium iodide (PI) Apoptosis Detection Kit was purchased from Abcam (Cambridge, UK). Bovine serum albumin (BSA) was from Serva (Heidelberg, Germany) and Triton™ X-100 was from Merck KGaA (Darmstadt, Germany). Anti-phospho-histone H2A.X (Ser^139^) rabbit primary antibody was from Cell Signaling Technology^®^ (Danvers, MA, USA) and Alexa Fluor^®^ 488 goat anti-rabbit IgG (H + L) secondary antibody was purchased from Thermo Fisher Scientific.

### 2.3. Cell Lines

Human glioblastoma cell line U87 was purchased from American Type Culture Collection (Rockville, MD, USA). The cell line was cultured in MEM supplemented with 10% FBS, L-glutamine (2 mM), 10,000 U/mL penicillin, 10 mg/mL streptomycin solution and 1% non-essential amino acids. Cells were maintained at 37 °C in humidified 5% CO_2_ atmosphere.

### 2.4. Primary Glioblastoma Culture

The histological grade of the surgical specimen from a patient with WHO grade IV glioblastoma was established by histopathological analysis. The tissue sample was collected during surgery and immediately processed. First, the tissue was chopped with a surgical blade in a sterile Petri dish. Chemical dissociation was achieved by adding Accumax solution (Sigma-Aldrich Chemie GmbH) to the chopped tissue for 15 min at room temperature. Dissociated tissue was then centrifuged and resuspended in 5 mL of DMEM/F12 medium, supplemented with 10% FBS, 2 mM L-glutamine, 10,000 U/mL penicillin, 10 mg/mL streptomycin and 25 µg/mL amphotericin B solution. DMEM/F12 medium was additionally supplemented with growth factors: 20 µL/mL B-27, 40 ng/mL epidermal growth factor and 20 ng/mL basic fibroblast growth factor, all purchased from Thermo Fisher Scientific. To confirm cell attachment, dissociated tissue was monitored for 48 h before changing the medium. Attached cells were grown until confluence prior to further studies. The glioblastoma origin of the established primary culture, named GBM-6, was confirmed as previously described [25]. The passage number of GBM-6 was kept low to prevent genetic and epigenetic changes in cells [28], to ensure the translational potential of obtained results [29].

### 2.5. MTT Assay

U87 and GBM-6 cells were seeded in triplicate into flat-bottomed 96-well cell culture plates at a density of 4000 cells per well and incubated overnight in 100 µL of the appropriate medium. The cells were treated for 72 h with increasing concentrations of Si306 or pro-Si306 (1, 2.5, 5, 10 and 25 µM) to assess cell growth. Cells cultured in media alone served as a negative control and 0.25% (v/v) DMSO was used as a solvent control. This colorimetric assay is based on the 3-(4,5-Dimethyl-2-thiazolyl)-2,5-diphenyl-2H-tetrazolium bromide (MTT) enzymatic reduction into formazan dye by active mitochondria in viable cells, indicating their metabolic activity. After treatment, 100 μL of MTT solution (2 mg/mL) was added to each well and the plates were incubated at 37 °C for 4 h. Subsequently, the formazan crystals formed in cells with viable mitochondria were dissolved in 200 µL of DMSO, and absorbance was measured at 540 nm using an automated microplate reader (LKB 5060–006 Micro Plate Reader, LKB, Vienna, Austria). The growth curves for Si306 and pro-Si306 were obtained by nonlinear regression analysis using GraphPad Prism 6.0 software (GraphPad Software, La Jolla, CA, USA).

### 2.6. Reactive Oxygen and Nitrogen Species Detection by Flow Cytometry

Reactive oxygen species and reactive nitrogen species (RNS) levels in U87 and GBM-6 cells were assessed by flow cytometry. DHE fluorescence was measured to detect superoxide anion levels in cells, while hydrogen peroxide and peroxynitrite anions were detected by DHR fluorescence. U87 and GBM-6 cells were seeded in 6-well plates at a density of 200,000 cells/well and incubated overnight. The cells were treated with 10 µM Si306 or pro-Si306 for 24 h. Next, adherent cells were harvested by trypsinization and incubated in with 1 µM DHE or DHR for 30 min at 37 °C in the dark. After washing twice in PBS, DHE and DHR fluorescence was measured on a CyFlow Space flow cytometer (Partec, Münster, Germany). in FL2 and FL1 channel, respectively. A minimum of 10,000 events were assayed for each sample. The results were analyzed by Summit 4.3 software (Dako Colorado Inc., Fort Collins, CO, USA).

### 2.7. Mitochondrial Transmembrane Potential Detection by Flow Cytometry

Mitochondrial transmembrane potential was evaluated by measuring JC-1 fluorescence on a flow cytometer. JC-1 is a cationic dye whose mitochondrial accumulation is dependent on the mitochondrial membrane potential [30]. The loss of mitochondrial transmembrane potential reduces the amount of red fluorescent JC-1 aggregates accumulated in mitochondria and increases the amount of cytoplasmic JC-1 monomers which fluoresce in green. U87 and GBM-6 cells were incubated overnight in 6-well plates at a density of 200,000 cells/well, and then treated with 10 µM Si306 or pro-Si306 for 24 h. The cells were then trypsinized, resuspended in 500 μL of appropriate media containing 2 μM JC-1 and incubated for 30 min at 37 °C in the dark. After washing twice in PBS, green and red fluorescence emissions were detected on a CyFlow Space flow cytometer and their ratio was analyzed in Summit 4.3 software. A minimum of 10,000 events were assayed for each sample.

### 2.8. Western Blot Analysis

The protein levels in GBM-6 and U87 cells were assessed via Western blot analysis. Cells were seeded into cell culture flasks (1 × 10^6^ cells per flask) and incubated overnight in 5 mL of appropriate media. Cells were then treated with 10 µM Si306 or pro-Si306 for 24 h. Next, the cells were lysed in Laemmli buffer (glycerol, 1M TRIS pH 6.8, 1% SDS, mQH2O and 20% β-mercaptoethanol) with bromphenol blue. Samples were then boiled for 5 min at 95 °C and kept at −80 °C until used. The proteins were first separated by 12% SDS-PAGE, and then transferred to PVDF membrane (Immobilon^®^-PSQ, Merck Millipore, Dublin, Ireland). The membranes were blocked with 5% non-fat dry milk (GE Healthcare, Buckinghamshire, UK) in TBST for 1 h at room temperature and incubated overnight at 4 °C with the following primary rabbit antibodies: polyclonal anti-Thioredoxin Reductase 1 (TrxR1) (Abcam; ab124954), polyclonal anti-Superoxide Dismutase 1 (SOD1) (Abcam; ab13498), polyclonal anti-Superoxide Dismutase 2 (SOD2) (Abcam; ab13533). After incubation with primary antibodies, the membranes were rinsed for 5 min, 6 times in TBST and incubated for 1 h at room temperature with a Horse Radish Peroxidase (HRP)-conjugated anti-rabbit secondary antibody (Abcam; ab6721). Immunoreactivity was detected by iBright™ CL1500 Western Blot Imaging System (Thermo Fisher Scientific). Each blot was re-probed with rabbit polyclonal anti-β-actin antibody (Abcam; ab8227) and incubated with (HRP)-conjugated anti-rabbit secondary antibody. All antibodies were diluted in TBST. Immunoreactive bands were quantified by densitometry using ImageJ software (U.S. National Institutes of Health, Bethesda, MD, USA) and expressed as relative values (i.e., density ratio normalized to the corresponding internal control, β-actin signal).

### 2.9. Double Strand DNA Breaks Detection by Flow Cytometry

The fluorescence intensity of anti-phospho-histone H2A.X antibody, which detects endogenous levels of phosphorylated H2A.X recruited to double strand DNA breaks sites, was used to quantify DNA damage in GBM-6 cells by flow cytometry. The cells were seeded at a density of 200,000 cells/well and grown overnight in 6-well tissue culture plates. The cells were subsequently treated with 10 µM Si306 or pro-Si306 for 72 h at 37 °C. After harvesting, the cells were washed in PBS and fixed in 4% paraformaldehyde for 10 min at room temperature. The cells were then permeabilized with ice cold 90% methanol for 30 min at 4 °C. After washing in PBS, the cells were blocked with 0.5% BSA in PBS for 1 h, and afterwards incubated overnight at 4 °C with anti-phospho-histone H2A.X antibody diluted 1:200 in 0.5% BSA in PBS. After washing in PBS, the cells were incubated for 30 min at room temperature with fluorescent Alexa Fluor 488 anti-rabbit IgG (H + L) secondary antibody diluted 1:1000 in 0.5% BSA in PBS. The cells were then washed and resuspended in 1 mL of PBS. The fluorescence intensity was measured in FL1 channel on a CyFlow Space flow cytometer. A minimum of 10,000 events were recorded for each sample. The collected data were analyzed using FCSalyzer 0.9.17 software (https://sourceforge.net/projects/fcsalyzer/, accessed on June 2019).

### 2.10. Double Strand DNA Breaks Detection by Immunocytochemistry

For immunostaining of phosphorylated H2A.X, GBM-6 cells were seeded (25,000 cells/chamber) in 4-well chamber slides (Nunc, Nalgene, Denmark) in 500 µL of appropriate medium and allowed to grow at 37 °C overnight. The cells were then treated for 72 h with 10 µM Si306 or pro-Si306. Next, the cells were washed in PBS, fixed in 4% paraformaldehyde for 15 min at room temperature and blocked in 0.5% BSA in PBS for 1 h. Anti-phospho-histone H2A.X antibody was applied at 1:1000 dilution in PBS/0.3% Triton X-100 and the cells were incubated overnight at 4 °C. After washing in PBS, fluorescent Alexa Fluor 488 anti-rabbit IgG (H + L) secondary antibody was applied at 1:1000 dilution in 0.5% BSA in PBS for 1 h at room temperature. To mark the nuclei, the cells were co-stained with hoechst 33342 and then mounted in Mowiol (Sigma-Aldrich Chemie GmbH). The cells were visualized under a Zeiss Axiovert inverted fluorescent microscope (Carl Zeiss, Jena, Germany) and imaged using AxioVision 4.8 software.

### 2.11. Cell Death Detection by Flow Cytometry

The percentages of apoptotic, necrotic and viable cells were assessed by Abcam Apoptosis Detection Kit through Annexin-V-FITC and PI staining according to the manufacturer’s instructions. GBM-6 cells were seeded and incubated overnight in 6-well culture plates at a density of 200,000 cells/well. GBM-6 cells were then treated for 72 h with 10 µM Si306, pro-Si306 or dasatinib. The attached and floating cells were collected by centrifugation and AV/PI staining of the cells was analyzed within 1 h by flow cytometry. The fluorescence intensity was measured in green FL1 and red FL2 channel on CyFlow Space flow cytometer. In each sample, 10,000 cells were recorded, and the percentages of viable (AV− PI−), early apoptotic (AV+ PI−), late apoptotic (AV+ PI+) and necrotic (AV− PI+) cells were analyzed by Summit 4.3 software.

### 2.12. β-Galactosidase Activity Assay

The β-galactosidase activity of senescent cells was assessed by flow cytometry by measuring the fluorescence of its substrate, fluorescein-di-β-D-galacto-pyranoside. GBM-6 cells were seeded in 6-well plates at a density of 200,000 cells/well and incubated overnight. The cells were then treated with 10 µM Si306 or pro-Si306 for 72 h. After treatment, adherent cells were trypsinized and suspended in 25 μL of appropriate media pre-warmed to 37 °C. FDG solution (2 mM) was prepared in sterile H_2_O, pre-warmed at 37 °C for 10 min, and 25 μL were added to each aliquot of cells. The cells and FDG solution were rapidly mixed and incubated for 1 min at 37 °C in a water bath. Next, 1 mL of ice-cold PBS with 0.2 μg of PI was added to the cells and they were stored on ice until analysis on a CyFlow Space flow cytometer. Dead cells were excluded from the analysis based on the uptake of PI. A minimum of 10,000 events were assayed for each sample. The results were analyzed by Summit 4.3 software.

### 2.13. Statistical Analysis

Statistical analysis was performed by GraphPad Prism 6.0 software. The flow cytometry results were analyzed by Student’s *t*-test, and the Western blot data were analyzed by two-way analysis of variance (ANOVA) test with Tukey’s post-hoc test, and the accepted level of significance was *p* < 0.05.

## 3. Results

### 3.1. Si306 and Pro-Si306 Induce Oxidative Stress in Glioblastoma Cells

**Figure 2:**
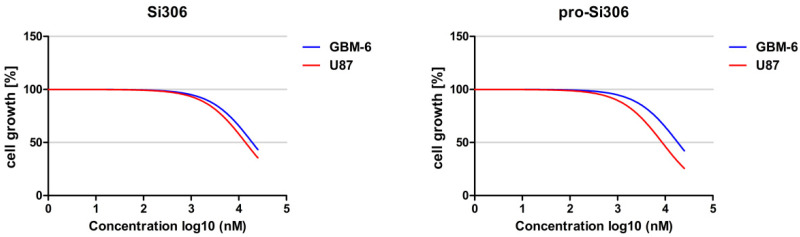
The sensitivity of glioblastoma cells to Si306 and pro-Si306. The effects of Si306 and pro-Si306 on cell growth inhibition were assessed by the MTT assay after 72 h. The graphs represent nonlinear regression fitted curves generated by GraphPad Prism 6.0 software.

For comparison of the compounds’ efficacy between U87 and GBM-6 cells, the treatment concentrations used in this study were selected based on the Si306 and pro-Si306 cell growth curves (Figure 2). The 10 µM concentration was efficient in inducing cell growth arrest in both commercial and patient-derived cells and was therefore selected for the oxidative stress studies.

The potential of Src tyrosine kinase inhibitors to generate ROS and RNS was analyzed by flow cytometry. The presence of superoxide anion, hydrogen peroxide and peroxynitrite anion was investigated in GBM-6 and U87 cells stained with DHE or DHR after 10 μM treatments with Si306 and pro-Si306 for 24 h.

**Figure 3:**
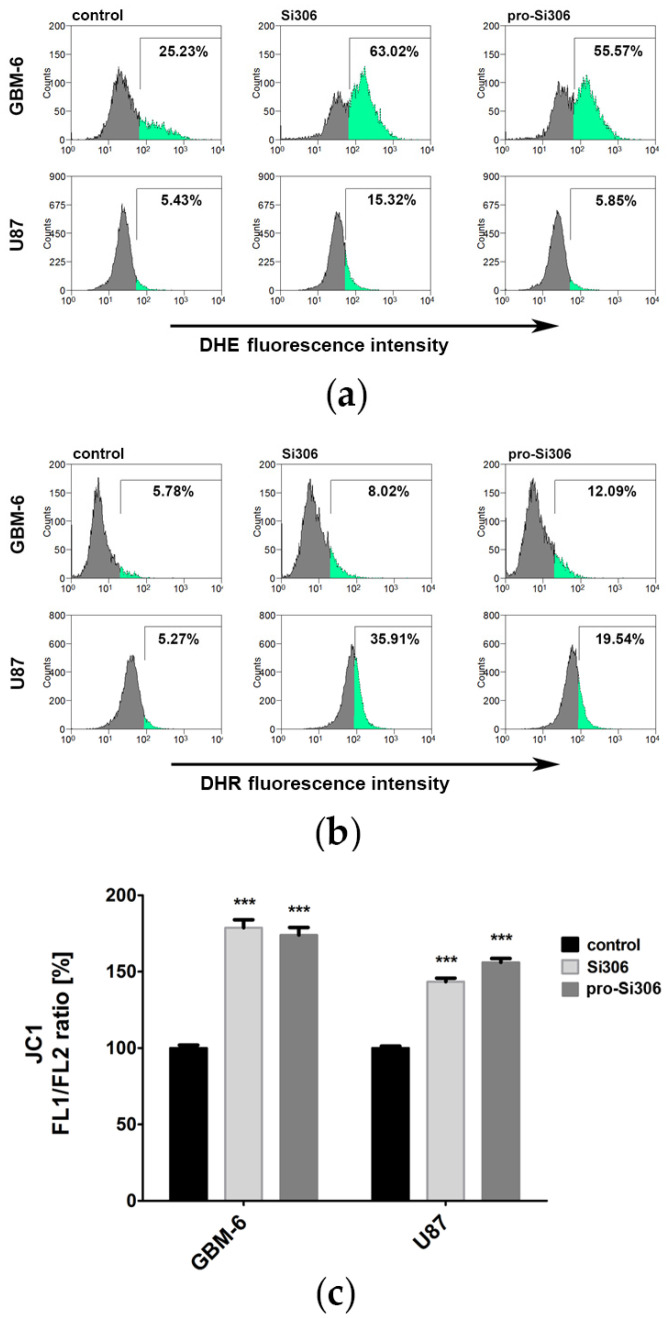
Si306 and pro-Si306 increase the levels of reactive oxygen and nitrogen species and depolarize mitochondrial membrane in glioblastoma cells. DHE, DHR and JC-1 fluorescence was evaluated by flow cytometry after 24 h treatment with 10 µM Si306 and pro-Si306. (**a**) Production of superoxide anion in GBM-6 and U87 cells. Percentages represent the number of cells with high levels of superoxide anions. The region of interest (green) was set to include approximately 5% of DHE-positive cells in the U87 control group. Due to the greater heterogeneity of GBM-6, the region of interest captured about 25% of DHE-positive control cells. (**b**) Production of hydrogen peroxide and peroxynitrite anion in GBM-6 and U87 cells. Percentages represent the number of cells with high levels of hydrogen peroxide and peroxynitrite anions. The region of interest (green) was set to include approximately 5% of DHR-positive cells in the control group. (**c**) Transmembrane potential of the mitochondrial membrane in GBM-6 and U87 cells. The histogram shows the relative ratio of JC-1 fluorescence in FL1 and FL2 channels. A statistically significant difference between treated and control groups is shown as *** (*p* < 0.001).

The intensity of DHE fluorescence, which is proportional to the O_2_^−^ levels, increased in GBM-6 cells after Si306 treatment producing 63.02% of DHE-positive cells compared to 25.23% recorded in the control sample (Figure 3a). Pro-Si306 increased the percentage of DHE-positive cells to 55.57%. In the U87 cell line, Si306 treatment increased the amount of superoxide anion from 5.43% to 15.32%, while pro-Si306 treatment had no effect (Figure 3a).

The intensity of DHR fluorescence, which is proportional to the H_2_O_2_ and ONOO^−^ levels, increased in Si306-treated GBM-6 cells, resulting in 8.02% of DHR-positive cells compared to the control, which had 5.78% (Figure 3b). Pro-Si306 treatment produced 12.09% DHR-positive cells. The U87 cell line displayed a significant increase in hydrogen peroxide and peroxynitrite levels after treatments with Src tyrosine kinase inhibitors. The highest percentage of DHR-positive cells was recorded after Si306 treatment, 35.91% compared to the control, which had 5.27%. After prodrug treatment, the percentage of DHR-positive cells was 19.54% (Figure 3b).

### 3.2. Si306 and Pro-Si306 Cause Depolarization of Mitochondria in Glioblastoma Cells

Since elevated ROS and RNS production influence mitochondrial function, the effect of Src tyrosine kinase inhibitors on mitochondrial membrane potential in GBM-6 and U87 cells after 24 h treatment was studied by flow cytometry (Figure 3c). Mitochondrial depolarization was determined by calculating the relative ratio of JC-1 green and red fluorescence.

In GBM-6 cells treated with 10 μM Si306 and pro-Si306, the relative ratio of green to red fluorescence of JC-1 increased by approximately 1.7-fold compared to the control, indicating a substantial number of cells with decreased mitochondrial transmembrane potential (Figure 3c). In the U87 cell line, Si306 and pro-Si306 treatments also efficiently depolarized the mitochondrial membrane, resulting in a 1.5-fold increase in the green/red fluorescence ratio compared to the control (Figure 3c).

### 3.3. Si306 and Pro-Si306 Increase the Expression of SOD1 and SOD2 in Glioblastoma Cells

**Figure 4:**
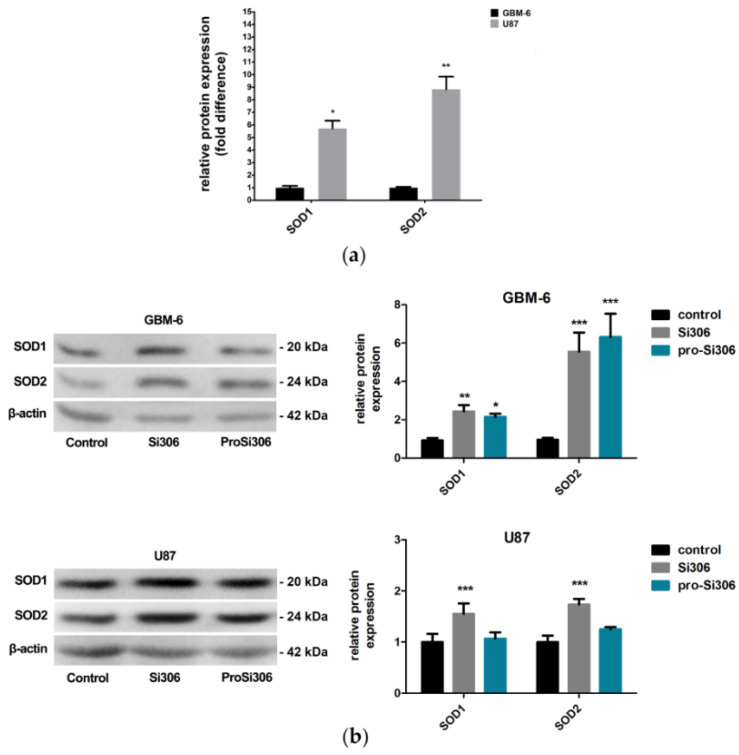
Si306 and pro-Si306 change the protein expression of antioxidant enzymes in glioblastoma cells. (**a**) Difference in protein expression levels of SOD1 and SOD2 between GBM-6 and U87 cells. The protein expression levels are presented as fold-difference compared to GBM-6 cells. The histogram represents Western blot data expressed as protein levels normalized to β-actin. Values are expressed as mean ± SD (*n* = 3). A statistically significant difference between GBM-6 and U87 cells is shown as * (*p* < 0.05) and ** (*p* < 0.01). (**b**) Representative Western blot images of superoxide dismutase 1 (SOD1) and superoxide dismutase 1 (SOD2) protein expression in GBM-6 and U87 cells after 24 h treatment with 10 µM Si306 and pro-Si306 are shown on the left panel. Quantification of protein expression levels of SOD1 and SOD2 in control cells and upon treatment is shown on the right panel. Histograms represent Western blot data expressed as protein levels normalized to β-actin. Values are expressed as mean ± SD (*n* = 3). A statistically significant difference between treated and control group is shown as * (*p* < 0.05), ** (*p* < 0.01) and *** (*p* < 0.001).

The anti-oxidative capacity of GBM cells was analyzed by Western blot. When compared to primary GBM cells, U87 cells displayed over 5-fold and 8-fold higher basal expressions of key antioxidant enzymes SOD1 and SOD2, respectively (Figure 4a).

To investigate whether the observed pro-oxidative effects of Si306 and pro-Si306 influence the expression of SOD1 and SOD2, GBM-6 and U87 cells were analyzed after 24 h treatment (Figure 4b). Src tyrosine kinase inhibitors increased the relative expression of SOD1 over 2-fold in GBM-6 cells, while SOD2 expression increased over 5-fold and 6-fold after treatment with Si306 and pro-Si306, respectively.

In U87 cells, the levels of SOD1 and SOD2 increased over 50% and 70% after Si306 treatment, respectively (Figure 4b). Thus, the effect of the prodrug on the expression levels of these enzymes in the U87 cell line after 24 h was not as evident as in primary cells.

### 3.4. Si306 and Pro-Si306 Induce DNA Damage in Primary Glioblastoma Cells

Since Src tyrosine kinase inhibitors induced prominent oxidative stress in GBM-6 cells, further investigations were focused on the effects resulting from elevated ROS production in primary GBM cells—DNA damage and cell death.

DNA damage in GBM-6 cells was analyzed by flow cytometry and fluorescence microscopy. The levels of phosphorylated H2A.X histone, a DNA double-stranded breaks marker, were determined after 72 h treatments with 10 μM Si306 and pro-Si306.

**Figure 5:**
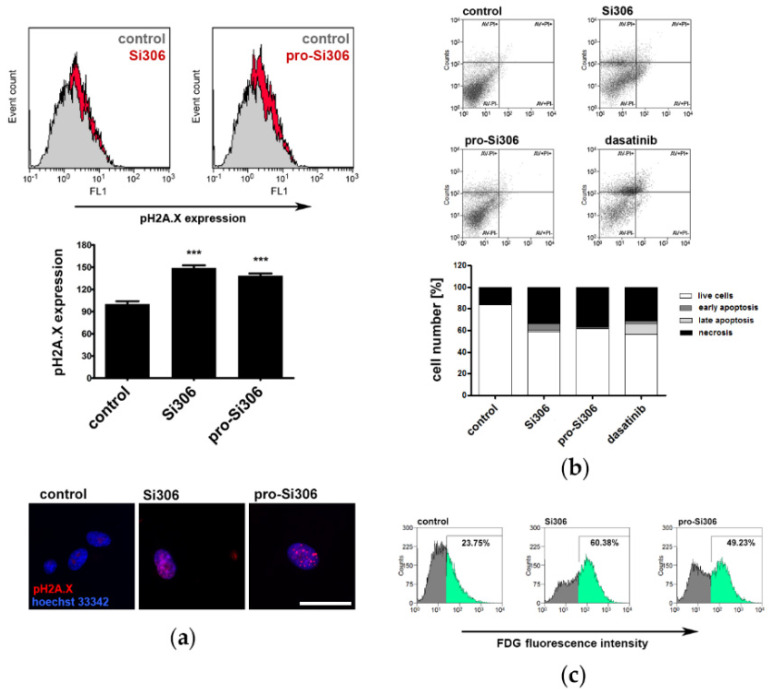
Si306 and pro-Si306 induce DNA damage, cell death and senescence in primary glioblastoma cells. (**a**) Representative flow-cytometric profiles of phospho-histone H2A.X (pH2A.x) expression in GBM-6 cells after 24 h treatment with 10 µM Si306 and pro-Si306 (upper panel). The histogram shows mean fluorescence intensity obtained by flow-cytometric detection of cells immunolabeled with pH2A.x antibody (middle panel). Values are expressed as mean ± SEM. Statistical significance between treated and control group is shown as *** (*p* < 0.001). Representative fluorescent micrographs of GBM-6 cells labeled with phospho-histone H2A.X antibody (red) and hoechst 33342 (blue) after 24 h treatment with 10 µM Si306 and pro-Si306 (bottom panel). Scale bar = 100 µm. (**b**) Cell death in GBM-6 cells was evaluated by AV/PI staining after 72 h treatment with 10 μM Si306, pro-Si306 and dasatinib. Representative flow-cytometric profiles of AV/PI stained cells for each condition are presented (upper panel). AV fluorescence was read on the FL1 green channel and PI fluorescence was read on the FL2 red channel. The histogram shows the percentage of viable (AV− PI−), early apoptotic (AV+ PI−), late apoptotic (AV+ PI+) and necrotic (AV− PI+) cells after treatment (bottom panel). (**c**) β-galactosidase activity in GBM-6 cells was evaluated by flow-cytometric measurement of FDG fluorescence after 48 h treatment with 10 µM Si306 and pro-Si306. Percentages in the representative flow-cytometric profiles show the number of senescent cells. The region of interest (green) was set to include approximately 20% of FDG-positive cells in the control group.

Src tyrosine kinase inhibitors caused DNA damage in GBM-6 cells to a similar extent. After Si306 treatment, double strand DNA breaks were detected in ~50% more cells compared to the control, while pro-Si306 treatment resulted in ~40% more cells with phosphorylated H2A.X histone staining (Figure 5a).

DNA damage in GBM-6 cells caused by Src tyrosine kinase inhibitors was also visualized on a fluorescence microscope after immunolabeling with phospho-histone H2A.X antibody (Figure 5a).

### 3.5. Si306 and Pro-Si306 Induce Necrosis in Primary Glioblastoma Cells

To examine the potential of Src tyrosine kinase inhibitors to induce cell death, GBM-6 cells were treated for 72 h. Flow-cytometric analysis of AV/PI-labeled GBM-6 cells showed that after Si306 application most cells entered necrosis—33.81%, while the number of GBM-6 cells in early and late apoptosis was 1.64% and 5.78%, respectively (Figure 5b). After 10 μM treatment with pro-Si306, 36.7% cells were necrotic. The effects of Si306 and pro-Si306 on cell death induction in primary GBM cells were similar to that of dasatinib, a well-known tyrosine kinase inhibitor. After dasatinib treatment, 31.25% of cells entered necrosis (Figure 5b).

### 3.6. Si306 and Pro-Si306 Induce Senescence in Primary Glioblastoma Cells

The potential of Src tyrosine kinase inhibitors to induce senescence, i.e., irreversible cell cycle arrest, in GBM-6 cells was also analyzed. The measurement of β-galactosidase activity by flow cytometry showed that Si306 treatment significantly increased the number of GBM-6 cells that metabolize FDG, 60.38% compared to 23.75% recorded in the control sample (Figure 5c). After pro-Si306 treatment, cellular senescence was detected in 49.23% of cells.

## 4. Discussion

Herein, we report the ability of two Src tyrosine kinase inhibitors to induce oxidative stress and cell death in glioblastoma cells. The effects of pyrazolo[3,4-*d*]pyrimidine derivative Si306 and its prodrug pro-Si306 on the induction of oxidative stress in human glioblastoma was evaluated in the U87 cell line and patient-derived GBM-6 cells. The reported findings present an addition to our previous studies, which demonstrated various effects of these compounds on glioblastoma in vitro and in vivo [22,25].

Cellular ROS and RNS levels may significantly affect the cancer response to therapy. The importance of ROS and RNS signaling in cancer is demonstrated by the development of new therapeutics that target the production of reactive oxygen species and nitrogen species, such as tyrosine kinase inhibitors and monoclonal antibodies [31]. Clinically relevant tyrosine kinase inhibitors such as afatinib [32], erlotinib [33], axitinib, imatinib [34] and numerous others have been reported to disrupt the redox homeostasis [31]. These receptor-targeted therapeutics have been shown to increase the oxidative stress in cancer cells up to a level that overpowers their antioxidant capacity. Excessive accumulation of ROS can lead to lipid peroxidation, protein oxidation, enzyme inactivation and oxidative DNA damage [35]. At the same time, ROS production has been shown to affect the mitochondrial membrane potential and permeability [36]. Mitochondria are the most important source of ROS, as their increased production can induce necrosis [37] and senescence [38]. Our results showed that Si306 and its prodrug caused oxidative stress in primary GBM-6 and U87 cells via increased O_2_^−^ production. It is important to highlight that oxidative stress triggered by Src tyrosine kinase inhibitors was considerably greater in primary GBM cells. In the U87 cell line, Si306 and pro-Si306 treatments were also accompanied by the generation of H_2_O_2_ and ONOO^−^. In line with other Src tyrosine kinase inhibitors shown to increase oxidative stress [15], the changes in ROS and RNS levels detected in Si306- and pro-Si306-treated GBM cells after 24 h are likely connected to inhibition of the Src pathway. As we previously reported, the 24 h treatment with these compounds significantly reduced Src activity in GBM-6 and U87 cells [25].

The elevated levels of ROS and RNS frequently activate signaling pathways that regulate the expression of antioxidant enzymes. SOD enzymes control the levels of ROS and RNS and as a result limit their potential toxicity and control numerous cellular processes regulated by their signaling functions. The Cu/Zn SOD (SOD1) is the cytoplasmic isoform but has also been found in lysosomes, peroxisomes, the nucleus and the mitochondrial intermembrane space, while the Mn SOD (SOD2) is localized in the mitochondrial matrix [39]. Since superoxide dismutase enzymes catalyze the conversion of superoxide anion into oxygen and hydrogen peroxide [39], the difference between detected reactive oxygen and nitrogen species in primary GBM cells and U87 cells after 24 h of treatment is likely due to the different amounts and activity of these enzymes. Specifically, the low amount of hydrogen peroxide detected in GBM-6 cells could be the consequence of a slower conversion from superoxide anions by the SOD enzymes. SOD1 and SOD2, more abundantly expressed in U87 cells, efficiently and more promptly converted the excess ROS generated by the Si306 and pro-Si306 treatments, resulting in more accumulated RNS compared to GBM-6 cells. Considering that GBM-6 cells have low basal expression of antioxidant enzymes, the expression of SOD1 and SOD2 in these cells increased in order to scavenge the excess ROS and minimize oxidative damage resulting from Si306 and pro-Si306 treatment.

To survive, cancer cells are under continuous pressure to sustain a balance between ROS levels and the response to oxidative stress [40]. In highly heterogeneous GBM tumors, different tumor cells survive a wide range of oxygen concentrations in their surroundings and adapt to their microenvironment through different mechanisms [40]. However, IDH1-mutated secondary GBMs are characterized by high oxidative burden and low antioxidative capacity [41,42,43,44], which makes them vulnerable to agents that increase ROS levels, such as Src tyrosine kinase inhibitors. High heterogeneity of GBM-6 cells regarding their ROS content reflects heterogeneity of cellular antioxidative capacity, which likely contributes to their strong sensitivity to Si306 and pro-Si306.

Treatments with Src tyrosine kinase inhibitors destabilized mitochondria and influenced mitochondrial membrane potential in GBM-6 and U87 cells. Changes in mitochondrial membrane potential in treated GBM cells could be related to higher O_2_^−^ levels, since the production of this anion can alter the permeability of the inner membrane of the mitochondria, causing mitochondrial membrane permeability transition [45]. Consequently, mitochondrial membrane permeability transition leads to loss of proton gradient and blockade of ATP genesis via oxidative phosphorylation. As a result, the permanent opening of the pores on the mitochondria contributes to the appearance of necrosis [46].

ROS and RNS can induce single- and double-stranded breaks on the DNA molecule, which frequently lead to cell death [47,48]. The phosphorylated form of histone H2A.X is often found at the site of DNA molecule repair and represents a marker of DNA damage. As revealed by phosphorylated histone H2A.X analysis, Si306 and pro-Si306 caused evident DNA damage in primary glioblastoma. A study showed that TK inhibitor axitinib induced double-stranded breaks on the DNA molecule, which caused cell cycle interruption and induction of senescence that ultimately led to necrosis [49]. Considering that Si306 and pro-Si306 cause DNA damage, it is possible that these TK inhibitors exert their effect in a similar manner.

We observed that Si306 and pro-Si306 led to necrosis in patient-derived cell culture. Furthermore, pro-Si306 showed higher efficacy compared to the parent drug. Glioblastoma is characterized by the presence of a necrotic core with hypoxic conditions [50]. These conditions promote the invasion of cells into healthy surrounding tissue with sufficient nutrients and oxygen. In this context, necrosis has a positive effect on the progression of glioblastoma. However, treatment of glioblastoma cells with Src tyrosine kinase inhibitors induced necrosis preceded by cellular senescence. Cellular senescence is characterized by several structural and functional features, including loss of mitotic activity, which lead to the interruption of cell division and eventually necrosis. Several anticancer drugs that target senescence are already in clinical trials [38]. An earlier study of the tyrosine kinase inhibitor axitinib’s effects on glioblastoma cells showed a similar mechanism of anticancer activity [49], suggesting that treatment-induced necrosis is a favorable outcome and that induction of senescence preceding necrosis could represent a potential strategy in the fight against glioblastoma. It is important to highlight that patient-derived GBM-6 cells proved to be highly prone to senescence and necrosis after treatment with Si306 and its prodrug. Therefore, necrosis induced by Si306 and pro-Si306 is an encouraging result in terms of glioblastoma treatment.

## 5. Conclusions

The overall results of this study suggest that the mechanism of action of the new pyrazolo[3,4-*d*]pyrimidine derivative Si306 and its prodrug is the induction of senescence and necrosis due to ROS production and DNA damage, in addition to the already demonstrated anti-invasive effects in vitro and in vivo [25]. It is noteworthy that pro-Si306 showed similar or greater efficacy compared to its parent drug, which is a rare feature. Although the effect of Si306 and pro-Si306 on necrotic cell death induction in primary glioblastoma was similar to dasatinib, the advantage of Si306 and its prodrug over dasatinib is their more favorable pharmacokinetic profile [22]. The anti-glioblastoma effects of Si306 and pro-Si306 demonstrated in this study, as well as previously obtained results in vitro and in vivo, particularly the ability of these TK inhibitors to cross the blood–brain barrier, give prospects for clinical trials of these compounds as potential candidates for glioblastoma therapy. The particularly strong efficacy of Si306 and pro-Si306 in patient-derived glioblastoma culture implies a promising translational potential for these novel Src tyrosine kinase inhibitors.

## Data Availability

The data presented in this study are available on request from the corresponding author. The data are not publicly available due to lack of institutional online database.

## References

[1] Bondy M.L., Scheurer M.E., Malmer B., Barnholtz-Sloan J.S., Davis F.G., Il’yasova D., Kruchko C., McCarthy B.J., Rajaraman P., Schwartzbaum J.A. (2008). Brain tumor epidemiology: Consensus from the Brain Tumor Epidemiology Consortium. Cancer.

[2] Stupp R., Mason W.P., van den Bent M.J., Weller M., Fisher B., Taphoorn M.J., Belanger K., Brandes A.A., Marosi C., Bogdahn U. (2005). Radiotherapy plus concomitant and adjuvant temozolomide for glioblastoma. N. Engl. J. Med..

[3] Wen P.Y., Kesari S. (2008). Malignant gliomas in adults. N. Engl. J. Med..

[4] Kannaiyan R., Mahadevan D. (2018). A comprehensive review of protein kinase inhibitors for cancer therapy. Expert Rev. Anticancer Ther..

[5] de Witt Hamer P.C. (2010). Small molecule kinase inhibitors in glioblastoma: A systematic review of clinical studies. Neuro Oncol..

[6] Woodworth G.F., Dunn G.P., Nance E.A., Hanes J., Brem H. (2014). Emerging insights into barriers to effective brain tumor therapeutics. Front. Oncol..

[7] Yeatman T.J. (2004). A renaissance for SRC. Nat. Rev. Cancer.

[8] Ahluwalia M.S., de Groot J., Liu W.M., Gladson C.L. (2010). Targeting SRC in glioblastoma tumors and brain metastases: Rationale and preclinical studies. Cancer Lett..

[9] Stettner M.R., Wang W., Nabors L.B., Bharara S., Flynn D.C., Grammer J.R., Gillespie G.Y., Gladson C.L. (2005). Lyn kinase activity is the predominant cellular SRC kinase activity in glioblastoma tumor cells. Cancer Res..

[10] Du J., Bernasconi P., Clauser K.R., Mani D.R., Finn S.P., Beroukhim R., Burns M., Julian B., Peng X.P., Hieronymus H. (2009). Bead-based profiling of tyrosine kinase phosphorylation identifies SRC as a potential target for glioblastoma therapy. Nat. Biotechnol..

[11] Dumont R.A., Hildebrandt I., Su H., Haubner R., Reischl G., Czernin J.G., Mischel P.S., Weber W.A. (2009). Noninvasive imaging of alphaVbeta3 function as a predictor of the antimigratory and antiproliferative effects of dasatinib. Cancer Res..

[12] Han X., Zhang W., Yang X., Wheeler C.G., Langford C.P., Wu L., Filippova N., Friedman G.K., Ding Q., Fathallah-Shaykh H.M. (2014). The role of Src family kinases in growth and migration of glioma stem cells. Int. J. Oncol..

[13] Lewis-Tuffin L.J., Feathers R., Hari P., Durand N., Li Z., Rodriguez F.J., Bakken K., Carlson B., Schroeder M., Sarkaria J.N. (2015). Src family kinases differentially influence glioma growth and motility. Mol. Oncol..

[14] Sun G., Kemble D.J. (2009). To C or not to C: Direct and indirect redox regulation of Src protein tyrosine kinase. Cell Cycle.

[15] Jin Y., Cai Q., Shenoy A.K., Lim S., Zhang Y., Charles S., Tarrash M., Fu X., Kamarajugadda S., Trevino J.G. (2016). Src drives the Warburg effect and therapy resistance by inactivating pyruvate dehydrogenase through tyrosine-289 phosphorylation. Oncotarget.

[16] Paech F., Bouitbir J., Krahenbuhl S. (2017). Hepatocellular Toxicity Associated with Tyrosine Kinase Inhibitors: Mitochondrial Damage and Inhibition of Glycolysis. Front. Pharmacol..

[17] Xue T., Luo P., Zhu H., Zhao Y., Wu H., Gai R., Wu Y., Yang B., Yang X., He Q. (2012). Oxidative stress is involved in Dasatinib-induced apoptosis in rat primary hepatocytes. Toxicol. Appl. Pharmacol..

[18] Schenone S., Radi M., Musumeci F., Brullo C., Botta M. (2014). Biologically driven synthesis of pyrazolo[3,4-d]pyrimidines as protein kinase inhibitors: An old scaffold as a new tool for medicinal chemistry and chemical biology studies. Chem. Rev..

[19] Schenone S., Brullo C., Musumeci F., Botta M. (2010). Novel dual Src/Abl inhibitors for hematologic and solid malignancies. Expert Opin. Investig. Drugs.

[20] Tintori C., Fallacara A.L., Radi M., Zamperini C., Dreassi E., Crespan E., Maga G., Schenone S., Musumeci F., Brullo C. (2015). Combining X-ray crystallography and molecular modeling toward the optimization of pyrazolo[3,4-d]pyrimidines as potent c-Src inhibitors active in vivo against neuroblastoma. J. Med. Chem..

[21] Calgani A., Vignaroli G., Zamperini C., Coniglio F., Festuccia C., Di Cesare E., Gravina G.L., Mattei C., Vitale F., Schenone S. (2016). Suppression of SRC Signaling Is Effective in Reducing Synergy between Glioblastoma and Stromal Cells. Mol. Cancer Ther..

[22] Fallacara A.L., Zamperini C., Podolski-Renic A., Dinic J., Stankovic T., Stepanovic M., Mancini A., Rango E., Iovenitti G., Molinari A. (2019). A New Strategy for Glioblastoma Treatment: In Vitro and In Vivo Preclinical Characterization of Si306, a Pyrazolo[3,4-d]Pyrimidine Dual Src/P-Glycoprotein Inhibitor. Cancers.

[23] Navarra M., Celano M., Maiuolo J., Schenone S., Botta M., Angelucci A., Bramanti P., Russo D. (2010). Antiproliferative and pro-apoptotic effects afforded by novel Src-kinase inhibitors in human neuroblastoma cells. BMC Cancer.

[24] Ceccherini E., Indovina P., Zamperini C., Dreassi E., Casini N., Cutaia O., Forte I.M., Pentimalli F., Esposito L., Polito M.S. (2015). SRC family kinase inhibition through a new pyrazolo[3,4-d]pyrimidine derivative as a feasible approach for glioblastoma treatment. J. Cell. Biochem..

[25] Nesovic M., Divac Rankov A., Podolski-Renic A., Nikolic I., Tasic G., Mancini A., Schenone S., Pesic M., Dinic J. (2020). Src Inhibitors Pyrazolo[3,4-d]pyrimidines, Si306 and Pro-Si306, Inhibit Focal Adhesion Kinase and Suppress Human Glioblastoma Invasion In Vitro and In Vivo. Cancers.

[26] Vignaroli G., Iovenitti G., Zamperini C., Coniglio F., Calandro P., Molinari A., Fallacara A.L., Sartucci A., Calgani A., Colecchia D. (2017). Prodrugs of Pyrazolo[3,4-d]pyrimidines: From Library Synthesis to Evaluation as Potential Anticancer Agents in an Orthotopic Glioblastoma Model. J. Med. Chem..

[27] Zawilska J.B., Wojcieszak J., Olejniczak A.B. (2013). Prodrugs: A challenge for the drug development. Pharmacol. Rep..

[28] Vogel T.W., Zhuang Z., Li J., Okamoto H., Furuta M., Lee Y.S., Zeng W., Oldfield E.H., Vortmeyer A.O., Weil R.J. (2005). Proteins and protein pattern differences between glioma cell lines and glioblastoma multiforme. Clin. Cancer Res. Off. J. Am. Assoc. Cancer Res..

[29] Ledur P.F., Onzi G.R., Zong H., Lenz G. (2017). Culture conditions defining glioblastoma cells behavior: What is the impact for novel discoveries?. Oncotarget.

[30] Reers M., Smiley S.T., Mottola-Hartshorn C., Chen A., Lin M., Chen L.B. (1995). Mitochondrial membrane potential monitored by JC-1 dye. Methods Enzymol..

[31] Mijatovic S., Savic-Radojevic A., Pljesa-Ercegovac M., Simic T., Nicoletti F., Maksimovic-Ivanic D. (2020). The Double-Faced Role of Nitric Oxide and Reactive Oxygen Species in Solid Tumors. Antioxidants.

[32] Truong T.H., Ung P.M., Palde P.B., Paulsen C.E., Schlessinger A., Carroll K.S. (2016). Molecular Basis for Redox Activation of Epidermal Growth Factor Receptor Kinase. Cell Chem. Biol..

[33] Shan F., Shao Z., Jiang S., Cheng Z. (2016). Erlotinib induces the human non-small-cell lung cancer cells apoptosis via activating ROS-dependent JNK pathways. Cancer Med..

[34] Chang S.P., Shen S.C., Lee W.R., Yang L.L., Chen Y.C. (2011). Imatinib mesylate induction of ROS-dependent apoptosis in melanoma B16F0 cells. J. Dermatol. Sci..

[35] Mallis R.J., Buss J.E., Thomas J.A. (2001). Oxidative modification of H-ras: S-thiolation and S-nitrosylation of reactive cysteines. Biochem. J..

[36] Chatterjee S., Kundu S., Bhattacharyya A. (2008). Mechanism of cadmium induced apoptosis in the immunocyte. Toxicol. Lett..

[37] Marnett L.J. (2000). Oxyradicals and DNA damage. Carcinogenesis.

[38] Pawlowska E., Szczepanska J., Szatkowska M., Blasiak J. (2018). An Interplay between Senescence, Apoptosis and Autophagy in Glioblastoma Multiforme-Role in Pathogenesis and Therapeutic Perspective. Int. J. Mol. Sci..

[39] Wang Y., Branicky R., Noe A., Hekimi S. (2018). Superoxide dismutases: Dual roles in controlling ROS damage and regulating ROS signaling. J. Cell Biol..

[40] Krawczynski K., Godlewski J., Bronisz A. (2020). Oxidative Stress-Part of the Solution or Part of the Problem in the Hypoxic Environment of a Brain Tumor. Antioxidants.

[41] Gilbert M.R., Liu Y., Neltner J., Pu H., Morris A., Sunkara M., Pittman T., Kyprianou N., Horbinski C. (2014). Autophagy and oxidative stress in gliomas with IDH1 mutations. Acta Neuropathol..

[42] Han S., Liu Y., Cai S.J., Qian M., Ding J., Larion M., Gilbert M.R., Yang C. (2020). IDH mutation in glioma: Molecular mechanisms and potential therapeutic targets. Br. J. Cancer.

[43] Tang X., Fu X., Liu Y., Yu D., Cai S.J., Yang C. (2020). Blockade of Glutathione Metabolism in IDH1-Mutated Glioma. Mol. Cancer Ther..

[44] Shi J., Sun B., Shi W., Zuo H., Cui D., Ni L., Chen J. (2015). Decreasing GSH and increasing ROS in chemosensitivity gliomas with IDH1 mutation. Tumour Biol. J. Int. Soc. Oncodev. Biol. Med..

[45] Cai J., Jones D.P. (1998). Superoxide in apoptosis. Mitochondrial generation triggered by cytochrome c loss. J. Biol. Chem..

[46] Wang K. (2014). Molecular mechanisms of liver injury: Apoptosis or necrosis. Exp. Toxicol. Pathol..

[47] Wang J.Y.J. (2019). Cell Death Response to DNA Damage. Yale J. Biol. Med..

[48] Wiseman H., Halliwell B. (1996). Damage to DNA by reactive oxygen and nitrogen species: Role in inflammatory disease and progression to cancer. Biochem. J..

[49] Morelli M.B., Amantini C., Nabissi M., Cardinali C., Santoni M., Bernardini G., Santoni A., Santoni G. (2017). Axitinib induces senescence-associated cell death and necrosis in glioma cell lines: The proteasome inhibitor, bortezomib, potentiates axitinib-induced cytotoxicity in a p21(Waf/Cip1) dependent manner. Oncotarget.

[50] Monteiro A.R., Hill R., Pilkington G.J., Madureira P.A. (2017). The Role of Hypoxia in Glioblastoma Invasion. Cells.

